# Comparison of the Efficacy of Midazolam and Dexmedetomidine As Sedative Agents in Third Molar Surgery

**DOI:** 10.7759/cureus.49477

**Published:** 2023-11-27

**Authors:** Alden S Jason, Gidean A Sundaram, Preethi J, Santhosh P Kumar, Murugesan Krishnan

**Affiliations:** 1 Oral and Maxillofacial Surgery, Saveetha Dental College and Hospitals, Saveetha Institute of Medical and Technical Sciences, Saveetha University, Chennai, IND; 2 Anesthesiology, Saveetha Dental College and Hospitals, Saveetha Institute of Medical and Technical Sciences, Saveetha University, Chennai, IND

**Keywords:** innovative, analgesia, minor oral surgery, third molar surgery, conscious sedation, dexmedetomidine, midazolam

## Abstract

Introduction

Minor dental and oral surgical procedures have been made comfortable with the rise in the use of daycare sedatives. Of these sedatives, midazolam is deemed a common sedative used for minor oral surgical procedures. Newer and safer sedatives such as dexmedetomidine have certain properties that may prove more efficient in oral surgical procedures. Third molar surgery is one of the most common minor oral surgical procedures performed in dentistry. Thus, this study aims to compare the efficacy of midazolam and dexmedetomidine as sedative agents in third molar surgery.

Materials and methods

Sixty young adult patients free from other comorbidities were included in the study with ages ranging between 18 and 50 years. The samples were matched for the difficulty of impacted teeth and randomly distributed among the groups. Groups were administered the respective sedative drugs midazolam and dexmedetomidine and their effects were observed through the Observer’s Assessment of Alertness/Sedation scale. The intraoperative vitals and sedation effects were checked every 15 minutes. Statistical analysis was done using IBM SPSS Statistics for Windows, Version 22 (Released 2013; IBM Corp., Armonk, New York, United States). Independent samples t-test and analysis of variance were the statistical tests employed to analyze the obtained data with p<0.05 considered as statistically significant.

Results

The depth of sedation has been both subjectively and objectively assessed and had no significant difference among the groups. The intra-operative heart rate assessment proved a more efficient reduction of pulse rate in the dexmedetomidine group as compared with the midazolam group. However, it was not statistically significant (p=0.121). The mean diastolic blood pressure showed a statistically significant difference between the groups with dexmedetomidine having lower blood diastolic pressure compared to midazolam (p=0.004). Quick arousal was witnessed in the dexmedetomidine group.

Conclusion

It can be concluded from the study that both dexmedetomidine and midazolam were equally effective as sedative agents for third molar surgery. However, the nature of cardio-protection, anti-sialagogue, and analgesic properties of dexmedetomidine can prove helpful, especially in minor oral surgical procedures like third molar surgery and it is recommended.

## Introduction

Minor dental and oral surgical procedures instill a feeling of fear and anxiety among people owing to the invasive nature of the procedure. Fear is said to be strongly dependent on the context and environmental stimuli and one’s response to that stimulus [1]. One of the pharmacological arsenals in the anxiety reduction protocol is the use of conscious sedation in minor oral surgical procedures. Conscious sedation is the one where the patient is able to maintain his or her respiration independently [2]. Conscious sedation has replaced the use of general anesthesia in the primary daycare setting [3]. Many guidelines and safety protocols in lieu of helping clinicians were published to explore the domain of conscious sedation [4].

Midazolam is a water-soluble, crystalline salt [5]. Midazolam is a benzodiazepine with a short duration of action and a plethora of uses namely in the reduction of anxiety and in helping with procedural amnesia. It contributes to a significant level of muscle relaxation while also causing sedation to the patient. Midazolam increases the affinity of gamma-aminobutyric acid (GABA) to the GABA receptor. GABA is one of the major inhibitory neurotransmitters of the central nervous system causing CNS depression. Benzodiazepines have no direct agonistic activity on GABA nevertheless it boosts the effect on the action of GABA [6].

Dexmedetomidine is a water-soluble midazole monohydrochloride [7]. Primarily dexmedetomidine is categorized as α-2 adrenoceptor agonist. Dexmedetomidine provides sedation, anxiolysis, hypnosis, amnesia, and analgesia. The nature of sedation is dose-dependent and highly documented in the literature [8]. Dexmedetomidine has been speculated to have the potential to be used even as an intravenous anesthetic agent. The amnestic effects of dexmedetomidine as well as the respiratory depression are diminished thereby providing a wide safety margin alongside the prevention of emergence confusion. The quick spontaneous arousal from a sleep-like state has been documented as the most significant property of dexmedetomidine [8].

The application of these two drugs for conscious sedation in the field of oral surgery has been well documented [4,7]. A direct comparison between the two widely used sedative agents for surgical removal of impacted teeth has been attempted in this study. Thus, this study aims to compare the efficacy of midazolam and dexmedetomidine as sedative agents in third molar surgery. The primary objective of the study was to evaluate the depth of sedation obtained with midazolam and dexmedetomidine drugs in third molar surgery. The secondary objectives of the study were to evaluate the changes in blood pressure and heart rate with the use of midazolam and dexmedetomidine drugs in third molar surgery.

## Materials and methods

Inclusion criteria

Patients who required surgical removal of impacted third molar teeth under the category American Society of Anesthesiologists (ASA) I and II as deemed fit by an anesthetist and were willing to participate were included in the study. Patients with similar types of impacted teeth (Pederson difficulty index - 6 - moderately difficult) and in the age range of 18-50 years irrespective of gender were considered for the study.

Exclusion criteria

Patients with any systemic co-morbidity and those deemed unfit for the procedure under sedation by the anesthetist were excluded. Any known allergy to the sedative agents and local anesthetic agents, and preoperative inflammation at the surgical site also warranted exclusion from the study.

Sample size calculation

Using the G*Power tool (G*Power software, latest version 3.1.9.7 (Heinrich-Heine-Universität Düsseldorf, Düsseldorf, Germany)), the mean and standard deviation of the previous studies were employed to arrive at the sample size. The calculated sample size for this study was n=60 with 30 participants in each group.

Procedure

After obtaining ethical committee clearance from the Institutional Human Ethical Committee - Saveetha Dental College (IHEC/SDC/OMFS-2205/22/232) and written informed consent from the patients, the study was conducted. The patients who were eligible were allocated into either of the two groups: group A (midazolam) or group B (dexmedetomidine) using a computer-generated model. The anesthetist's fitness was obtained, and patients were kept nil oral six hours prior to the procedure. An IV cannula was placed in the dorsum of the hand of every patient to administer the drug. Patients were administered midazolam 0.2mg/kg body weight or dexmedetomidine 1.5 mcg/kg through the intravenous route 10 minutes before the procedure. Continuous monitoring of the pulse rate, the systolic and diastolic blood pressure was done. Arrangements were made to give Inj. ketamine 0.5mg/kg if an escape sedative was warranted due to lack of sedation. Surgical removal of the impacted teeth was performed for all the patients by the same surgeon.

Outcome variables

The primary outcome variables were the depth of sedation as measured by the Observer’s Assessment of Alertness/Sedation (OAA/S) scale. Secondary variables included systolic and diastolic blood pressure and heart rate. The amount of drug delivered was recorded. Sedation variables, blood pressure, and heart rate were recorded 10 minutes after the medication was delivered. Henceforth, every 15 minutes throughout the procedure, two anesthesia technicians trained with the scales assessed the sedation status using the OAA/S scale. Heart rate and blood pressure were also documented every 15 minutes throughout the procedure. The surgeon, the participant, and the anesthesia technicians were unaware of the administered drug and were blinded.

Statistical analysis

Statistical analysis was done using IBM SPSS Statistics for Windows, Version 22 (Released 2013; IBM Corp., Armonk, New York, United States). The normality of the data was checked with the Shapiro-Wilk test. Independent sample t-test and analysis of variance (ANOVA) were employed with p<0.05 considered as statistically significant.

## Results

The descriptive statistics of our study population are depicted in Table 1. The average age in both groups was similar and the participants were young adults. This is representative of the majority of the population who undergo third molar surgery in this age group. The samples also matched according to the body weight with an overall mean body weight of 58.1 kg in the midazolam group and 59.5 kg in the dexmedetomidine group. The duration of the surgery in all the study samples was less than 90 minutes.

**Table 1:** Descriptive data of the sample population included in the study A: Midazolam group; B: Dexmedetomidine group; SD: Standard deviation; %: Percentage

Parameters	Group A	Group B
Mean age in years (SD)	26.9 (5.7)	27.1 (6.2)
Mean body weight in kilograms (SD)	58.1 (10.2)	59.5 (10.8)
Male: Female (%)	12 (40%): 18 (60%)	15 (50%): 15 (50%)
ASA physical status I (%)	28 (93.3%)	28 (93.3%)
ASA physical status II (%)	2 (6.7%)	2 (6.7%)
Mean duration of surgery in minutes (SD)	70.8 (16.1)	67.6 (15.4)
Number of escape sedatives administered	0	0

Sedation scores obtained in midazolam and dexmedetomidine group participants are depicted in Table 2 and Table 3, respectively. The depth of sedation was assessed every 15 minutes among the participants. The depth of sedation reduced significantly after 60 minutes from the administration of the drug in both groups. There was no significant difference elicited between the groups in terms of depth of sedation as assessed by the OAA/S scale and expressed in sedation scores with percentages in brackets.

**Table 2:** OAA/S scale score recorded at 15-minute intervals for group A participants Group A: Midazolam group; OAA/S scale: Observer’s Assessment of Alertness/Sedation scale; mins: Minutes

Score	Responses	15 mins	30 mins	45 mins	60 mins	75 mins	90 mins
5	No response to mild shaking	0	0	0	0	0	0
4	Response to mild shaking	4 (13.3)	5 (16.7)	10 (33.3)	0	0	0
3	Response to repeated name-calling	9 (30)	14 (46.7)	8 (26.7)	15 (50)	7 (23.3)	2 (6.7)
2	Response to name in a lethargic manner while spoken in a normal tone	11 (36.7)	11 (36.7)	12 (40)	15 (50)	8 (26.7)	10 (33.3)
1	Response to name in a swift manner while spoken in a normal tone	6 (20)	0	0	0	15 (50)	18 (60)

**Table 3:** OAA/S scale score recorded at 15-minute intervals for group B participants Group B: Dexmedetomidine group; OAA/S scale: Observer’s Assessment of Alertness/Sedation scale; mins: Minutes

Score	Responses	15 mins	30 mins	45 mins	60 mins	75 mins	90 mins
5	No response to mild shaking	0	0	0	0	0	0
4	Response to mild shaking	2 (6.7)	1 (3.3)	5 (16.7)	2 (6.7)	2 (6.7)	1 (3.3)
3	Response to repeated name-calling	4 (13.3)	2 (6.7)	6 (20)	19 (63.3)	8 (26.7)	14 (46.7)
2	Response to name in a lethargic manner while spoken in a normal tone	4 (13.3)	19 (63.3)	19 (63.3)	9 (30)	20 (66.7)	15 (50)
1	Response to name in a swift manner while spoken in a normal tone	20 (66.7)	8 (26.7)	0	0	3 (10)	0

**Figure 1:**
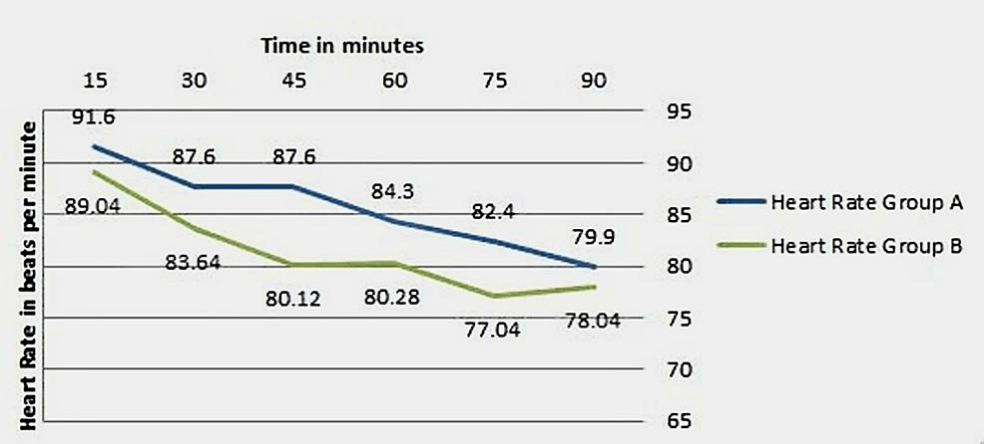
Heart rate measurements among the study participants during the study period Group A: Midazolam group; Group B: Dexmedetomidine group

The mean heart rate in group A was 85.5 beats per minute while that of group B was 81.3 beats per minute. The difference in mean heart rate between the two study groups was statistically not significant (independent sample t-test) (p=0.121) (Figure 1).

The mean systolic blood pressure was 120.5 mmHg in group A and 118.5 mmHg in group B. The mean diastolic blood pressure was 84.3 mmHg in group A and 77.4 mmHg in group B. The net difference in the systolic and diastolic blood pressure between the groups was subjected to statistical comparisons. One-way ANOVA was performed which showed a statistically significant difference in mean diastolic blood pressure (p=0.004) between the two study groups, while the mean systolic blood pressure was statistically not significant (p=0.356) between the two study groups (Table 4).

**Table 4:** Blood pressure measurements among the study participants during the study period Time in minutes BP: Blood pressure in mmHg; Group A: Midazolam group; Group B: Dexmedetomidine group; SD: Standard deviation

Time	Group A				Group B			
	Systolic BP		Diastolic BP		Systolic BP		Diastolic BP	
	Mean	SD	Mean	SD	Mean	SD	Mean	SD
15	123.34	5.2	89.1	4.1	118.72	4.7	79.34	5.6
30	118.89	4.5	86.59	7.3	116.96	7.4	79.04	4
45	125.6	6.2	87.32	6.2	120.35	9.4	78.7	5.1
60	120.8	4.3	82.41	9.7	119.24	8.1	73.2	10.6
75	122.6	4.3	80.43	3.9	118.31	7.2	76.31	4.3
90	111.8	8.2	79.8	9.9	117.64	4.7	77.72	2.9

## Discussion

This study compared the efficacy of intravenous dexmedetomidine versus intravenous midazolam for sedating adult patients undergoing minor oral surgical procedures like third molar surgery. Most of the studies reported in the literature are pertaining to the pediatric population in which mostly dexmedetomidine was compared to other sedative agents [3,4,9].

The depth of sedation in the study was assessed using the OAA/S scale besides the heart rate and the blood pressure which were measured to obtain the subjective and objective depth of sedation and reduction of anxiety. The baseline heart rate was 86 beats per minute on average. The sinus tachycardia could be a result of the anxiety of the patient as preoperative anxiolytics were omitted. Sporadically during the procedure heart rate rise was noticed just during times of surgical discomfort. Nevertheless, no intervention in lieu of the increased heart rate was warranted at any point of the surgical procedure.

According to our study, dexmedetomidine has proved very efficient in maintaining cardio-protective sedation similar to observations made by Mishra et al. [10]. Midazolam has established cardio-protective properties [11,12] which has also been reinforced in our study. The midazolam group showed similar depth of sedation as compared to the dexmedetomidine group at the 60-minute mark, with 50% of the group A (midazolam) samples and 63.3% of the group B (dexmedetomidine) samples scored a value of three in the OSA/A scale. At the 90-minute mark, 90% of group A (midazolam) expressed a response to name-calling in a normal tone while only 50% of the study population in group B (dexmedetomidine) responded. Thus, the depth of sedation achieved was almost similar in both groups at the 60-minute mark; however, the recovery from sedation was swift in the midazolam group at 90 minutes compared to the dexmedetomidine group.

The side effects of dexmedetomidine include blood pressure changes, nausea, and bradycardia. Besides these, it possesses analgesic properties and anti-sialagogue properties. Hence it can be considered ideal for dental procedures in accordance with other studies [12,13]. Midazolam is known to have a tendency to cause respiratory depression and induce nausea. However, such incidents were very limited as reported in previous literature [5,14], and were not encountered in the course of our study. With further research, the daycare sedation protocols could alleviate all potential anxiety surrounding dental treatments and make conscious sedation a safe treatment option. It can provide a better quality of life in patients undergoing minor oral surgical procedures irrespective of the difficulty involved in the surgical procedures and overall aiding in the perioperative management [15,16].

Limitations of the study

This would be the limited sample size employed in the study. The use of further scales of sedation to measure the depth of sedation could prove effective. The depth of sedation for minor oral surgical procedures other than third molar surgeries has not been assessed and can be included in future studies.

## Conclusions

It can be concluded from the study that both dexmedetomidine and midazolam were equally effective as sedative agents for third molar surgery. Due to the profound analgesic property and anti-sialagogue properties of dexmedetomidine, its use in dental and oral minor surgical procedures like third molar surgery is recommended.
